# Determinants of time to first marriage and birth intervals among women of child bearing age in Dabat Health and demographic surveillance system site, Northwest Ethiopia

**DOI:** 10.1371/journal.pone.0281997

**Published:** 2023-02-24

**Authors:** Nega Mihret Alazbih, Assefa Hailemariam Kaya, Mezgebu Yitayal Mengistu, Kassahun Alemu Gelaye

**Affiliations:** 1 Department of Population Studies, University of Gondar, Gondar, Ethiopia; 2 Population studies, Addis Ababa University, Addis Ababa, Ethiopia; 3 Department of Health System and Policy, Institute of Public Health, College of Medicine and Health Sciences, University of Gondar, Gondar, Ethiopia; 4 Department of Epidemiology and Biostatistics, Institute of Public Health, College of Medicine and Health Sciences, University of Gondar, Gondar, Ethiopia; University of Botswana, BOTSWANA

## Abstract

**Background:**

Event histories such as marriage and birth have been used to study fertility behavior of women. Understanding the timing of these events provide insight to reproductive patterns of the population. Thus, the aim of this study was to assess the timing of marriage and durations of birth intervals and their associated factors, and and to examine their effects on the current fertility among women in Dabat health and demographic surveillance system site, Northwest Ethiopia.

**Methods:**

A community based cross-sectional survey was carried out in the beginning of 2020 among 1649 women of reproductive age group. Data were collected using structured and interviewer administered questionnaire. The parametric survival analysis was employed to estimate the relationships among socioeconomic and demographic variables with outcome variables, the timing of age at first marriage and duration of birth intervals.

**Results:**

This study confirmed that median age at first marriage was the lowest estimated at 15 years which was below the national and regional average. The result of the study also revealed that married women waited almost a median duration of three years for their first, second, third and fourth child which was increased to nearly four years for three years preceding the survey. The parametric survival analysis showed woman’s education, occupation, and current age were the predictors of age at first marriage. divorce experience, women empowerment and marriage cohort were the determinant factors of first birth interval; women education, child death, and ideal number of children were the predictors of second and third birth intervals; and media exposure and child death experience of women were predictors of fourth birth interval.

**Conclusion:**

The study indicated that median age at first marriage was the lowest though the successive birth intervals were longer. The survival analysis identified women’s education, occupation, child death and ideal number of children affected the timing of age at first marriage and duration of birth intervals. Hence, encouraging women for higher education and giving opportunity to women in employments may contribute for delaying age at first marriage and increasing the duration of birth intervals which in turn slowing down the fertility of women.

## Background

Event histories such as marriage and birth have been used to study fertility behavior of women [1]. Marriage has traditionally been early and universal in sub-Saharan Africa and this has been blamed for high fertility [2]. The shift from arranged to love marriage since recent times due to changes in socio-cultural setting, particularly the rapid increase in formal education eliminates parental roles in making decisions on the timing of marriage and child bearing [3, 4]. Marriage that is based on self-selection of a partner takes longer time to search for “appropriate” mate that leads to later marriage [5]. In addition, the recent directions of the developing nations to delay first marriages for women which would normally increase the age at first birth are also the basics of some of the most dramatic shifts in demographic patterns including fertility reduction [6]. However, Ethiopia has still one of the highest rates of early marriage among Sub–Saharan African countries, though the country’s revised family code Proclamation of 2000 states that the minimum legal age for marriage is 18 for both sexes [7]. The percentage of women with reproductive age marrying before age 18 has showed a slight declined from 63% in 2011 to 58% in 2016 [8], and it was highest in Amhara region, 85% in 2011 [9].

Early marriage is often associated with early childbearing that marks a woman’s transition into motherhood [10, 11]. The age at first birth also plays a significant role in the future life of each individual woman and has a direct relationship with fertility [12]. In the absence of successful fertility control, the timing of a woman’s first birth affects the number of children she bears throughout her reproductive period, and women who give birth early in life have more children than women who give birth later [13]. The 2016 Ethiopian Demographic and Health Survey revealed that median age at first marriage and birth among women age 20–49 constituted 17.5 and 19.7 respectively. The same source also revealed that there was a great variation by region and are below the national figure in Amahara National Regional State (ANRS) at 16.2 and 19.4 in 2016 respectively. But median interval between first marriage and first birth was shorter in Tigray, Oromia, Somalia, Benishangul-Gumuz, and Gambela estimated at 2.4, 1.7, 1.9, 1.8 and 2.1 years with 17.2, 17.4, 18.1, 17.1 and 17.3 age at first marriage respectively.

Birth interval, the time duration between two consecutive live births, is also one of the important indicators of fertility situation of a country as the reproductive preference for spacing instead of limiting is peculiar to sub-Saharan Africa [14, 15]. A study in sub-Saharan Africa revealed the widespread desire of women to have longer birth intervals than they are currently having in the region. Between a quarter and a third of women reported that they had recently experienced a birth sooner than they wanted [14]. Thus, birth intervals experienced by women could provide a more comprehensive picture of the dynamics of fertility situations [1]. A mother is able to conceive the next child sooner, possibly leading to a shorter birth interval and higher fertility. On the other hand, longer intervals between consecutive births decrease the number of children a woman can have [15]. The desire to lengthen birth intervals could lower the birth rate that accelerates the fertility transition [14]. In Ethiopia like many other Sub-Saharan African countries, fertility is still high, though showed a declining trend. Total fertility Rate (TFR) in the country and in ANRS were 4.6 and 3.7 children per woman respectively in 2016 [8]. The TFR in the study area, Dabat Health and Demographic Surveillance System site (HDSSs) was also observed a slight decreasing from 4.4 in 2009 to 4.2 births per woman in 2012 [16].

Birth interval has also received attention in public health researches because of its implications for maternal and child health [17]. Globally, a birth interval of fewer than 18 months is associated with increased risk for neonatal mortality, infant mortality, under-five mortality, and maternal mortality [18]. It has been estimated that 1.6 million deaths of under-five would be averted annually if all birth to pregnancy intervals were spaced at least 3 years [19]. To reduce the risk of adverse maternal and child health outcomes, the most recent World Health Organization (WHO) recommendation for a healthy pregnancy interval is at least two years (24 months) or a birth-to-birth interval of 33 months [20]. However, it was estimated globally that 25% of births still occur at intervals less than 24 months [21]. The median length of birth interval in Ethiopia is 34.5 months with high percentage of births occurring after an interval of less than 36 months in the country and in ANRS, 54% and 33.5% respectively in 2016 [8]. The same source also showed that Ethiopia still experiences higher rates of maternal, neonatal, and infant mortality of 412/100,000, 30/1000, and 48 per 1000 live births, respectively in 2016.

As noted elsewhere, in a population where fertility often takes place within marriage and contraceptive practice is low, there is an inverse relationship between age at first marriage and fertility. However, total fertility rate of ANRS is the lowest in the country with low age at first marriage [8]. It is also indicated that there is a wider gap between age at first marriage and first birth (long first birth interval) in ANRS compared to other regions in the country with a median interval of 3.2 years. Hence, there is a need to investigate why fertility is becoming very low with low age at first marriage and birth, and why the interval between first marriage and birth is longer in the population of the region that experiences relatively lower contraceptive use that would provide insight to reproductive patterns which is critical for the country with a population policy aiming at reducing fertility. Thus, this study was aimed to assess the timing of marriage and durations of birth intervals and their associated factors among women in Dabat health and demographic surveillance system site, HDSSs, Northwest Ethiopia.

## Methods

### Study setting and design

A community based cross-sectional survey was employed from January 10 to February 29, 2020 at Dabat HDSSs. The Dabat HDSSs is hosted by the University of Gondar located in Dabat district, Northwest Ethiopia, 75 Kms north of Gondar and 804 Kms northwest of Addis Ababa. Dabat district has a total of 26 rural and 4 urban kebeles (the smallest administration unit in Ethiopia). According to the 2007 census report, the district had an estimated total population of 145,458 which was projected to be 172, 836 in 2015 [22, 23].

Dabat district was initially selected purposively as a surveillance site for its unique three agro-climatic zones (lowland, midland and highland) with altitude ranging from about 1000 to 2500 meters above sea level [16]. Currently DHDSSs incorporates thirteen kebeles. Three of these kebeles were included in the surveillance site in 2014 and these were not included in this study. Out of ten kebeles, seven were from rural and three from urban kebeles, among which seven were from highland, one from midland and two from lowland areas, sampled by using probability proportional to size technique. The study population included women with reproductive age who have been living in the surveillance site.

### Sample size and sampling techniques

The study population included women of reproductive age who have been living in the surveillance site at the time of the interview. The sample size was determined using a single population proportion formula considering the following assumptions: the proportion of women with reproductive age who had a first birth by age greater than 20 (47%) [8]; the age at which childbearing commences is an important determinant of the overall level of fertility, with 95% level of confidence, 2.5% margin of error, and 10% non-response rate. Consequently, the required sample size was determined to be 1683. The sample size is proportionally allocated to each of the Kebele administrations based on their size of women of reproductive age population. To select the study subjects, women with reproductive age, first, the list of women of reproductive age with kebele, household in which at least one woman with reproductive age has been living, and individual identifier was prepared from the database. Then after, the allocated sizes of women of reproductive age were selected using a simple random sampling technique using a random number generator. The Kebele, household, and individual identifiers were used to locate the selected women for an interview. When there were two or more women of reproductive age from the same family living in the same home, one of them was selected by lottery method and interviewed.

### Data collection tools and procedures

Data were collected using structured, pretested, and interviewer administered questionnaire. The questionnaire was adopted from Ethiopian demographic and health survey and other literatures. It consists of sociodemographic and reproductive health characteristics of the respondents. To maintain consistency, the questionnaire was first translated from English to Amharic, the native language of the study area, and was retranslated back to English by professional translators and demographers. The tool was pretested on 6% (100) of the total sample out of the study area. During pretest, the acceptability and applicability of the procedures and tools were evaluated. Twenty data collectors, two in each Kebele, and five field supervisors, both working in DHDSS site, were recruited for the study. Two days training on the objective of the study, content of the questionnaire, confidentiality of information, how to use open data kit (ODK) software suites and techniques to conduct interview was given to data collectors and supervisors. Data were collected using ODK software suites which is one of the most well-known mobile data collection frameworks. It is capable of controlling data entry error with its skip rules, internal consistency checks and facilities such as supporting multiple languages and making calculations [24]. Data completeness was checked by supervisors, data manager of Dabat research center, and principal investigator during data collection.

#### Study variables

The dependent variables were the timing of age at first marriage (measured in years) and durations of successive birth intervals (measured in months). The duration between first marriage and first live birth is considered first birth interval. Other intervals are calculated by the difference between two successive live births. The events for this study are the first union with a husband, first birth, second birth, third birth and fourth birth. For women who have never been married and did not give birth at any birth interval (censored), the time was measured till the date of the interview. The independent variables were the socioeconomic and demographic characteristics of women with reproductive age. The variables were categorized for analysis as shown in Tables 1 and 2.

**Table 1:** Proportion ever-married, median ages at first marriage in year, and median birth intervals in month, in Dabat HDSSs, Ethiopia, 2020.

Characteristics	Categories	Total women	Ever married women(n = 924)		Birth intervals (in months)							
		(n = 1649)			First birth interval (n = 783)		Second birth interval (n = 817)		Third birth interval (n = 695)		Fourth birth interval (n = 554)	
		No. (%)	No. (%)	Median AFM*	No. (%)	Median	No. (%)	Median	No. (%)	Median	No. (%)	Median
Current Residence	Urban	587 (35.6)	303 (32.8)	18	257 (32.8)	36	258 (35.6)	49.5	191 (27.5)	40	122 (22)	43
	Rural	1062 (64.4)	621 (67.2)	15	526 (67.2)	43	559 (68.4)	35	504 (72.5)	35	432 (78)	36
Current age	15–24	775 (47)	145 (15.7)	16	132 (16.9)	29	93 (11.4)	33	36 (5.2)	21.5	7 (1.3)	14
	25–34	400 (24.26)	333 (36)	15	272 (34.7)	39	298 (36.5)	38.5	246 (35.4)	36	164 (29.6)	37
	35+	474 (28.74)	446 (48.3)	15	379 (48.4)	51	426 (52.1)	36	413 (59.4)	36	383 (69.1)	37
Age at first marriage	< 18		662 (71.6)	14	587 (75)	47	607 (74.3)	37	551 (79.3)	36	460 (83)	37
	≥ 18		262 (28.4)	19	196 (25)	28	210 (25.7)	35.5	144 (20.7)	36.5	94 (17)	36
Age at first birth	< 18	352 (40.1)	343 (41)	14			339 (41.5)	36	310 (44.6)	36	259 (46.8)	37
	≥ 18	525 (59.9)	493 (59)	17			478 (58.5)	36	385 (55.4)	35	295 (53.2)	36
Place of birth	Rural	1355 (82.2)	802 (86.8)	15	684 (87.4)	43	714 (87.4)	35	629 (90.5)	36	516 (93.1)	36
	Urban	294 (17.8)	122 (13.2)	18	99 (12.6)	26	103 (12.6)	52	66 (9.5)	49.5	38 (6.9)	49
Educational Level	None	584 (35.4)	523 (56.6)	14	442 (56.5)	51.5	486 (59.5)	35	467 (67.2)	36	416 (75.1)	36
	Primary	435 (26.4)	166 (18)	15	141 (18)	36	143 (17.5)	37	111 (16)	34	79 (14.3)	36
	Secondary+	630 (38.2)	235 (25.4)	18	200 (25.5)	30	188 (23)	47.5	117 (16.8)	49	59 (10.6)	43
Media exposure	Yes	459 (27.8)	212 (22.9)	18	178 (22.7)	32	179 (21.9)	49	125 (18)	45	79 (14.3)	46
	No	1190 (72.2)	712 (77.1)	15	605 (77.3)	43	638 (78.1)	35	570 (82)	35	475 (85.7)	36
Occupation	Housewife	776 (47.1)	696 (75.3)	15	588 (75.1)	43	643 (78.7)	35	584 (84)	35	492 (88.8)	36
	Student	576 (34.9)	41 (4.4)	16	38 (4.9)	33.5	20 (2.5)	25.5	5 (0.7)	48	3 (0.5)	49
	Employed	168 (10.2)	129 (14)	19	109 (13.9)	30	112 (13.5)	55	79 (11.4)	43	44 (8)	48
	Job seekers	129 (7.8)	58 (6.3)	18	48 ()	31	42 (5.1)	56.5	27 (3.9)	56	15 (2.7)	38
Divorce before 1^st^ birth	Yes		138 (15)	14	137 (17.5)	67						
	No		786(85)	15	646 (82.5)	37						
Divorce experience	Yes		215 (23.3)	15			188 (23)	41.5	158 (22.7)	36	129 (23.3)	39
	No		709 (76.7)	15			629 (77)	35	537 (77.3)	36	425 (76.7)	36
Child death experience	Yes	102 (11.6)	97(11.6)	14	90 (11.5)	49.5	94 (11.5)	33.5	90 (12.9)	32	84 (15.2)	33.5
	No	775 (88.4)	739(88.4)	15	693 (88.5)	40	723 (88.5)	37	605 (87.1)	36	470 (84.8)	38
Husband’s education	None		536 (58.0)	15	444 (56.7)	49	488 (59.7)	35.5	451 (64.9)	36	392 (70.8)	37
	Primary		166 (18.0)	15	148 (18.9)	40	147 (18)	35	126 (18.1)	36	101 (18.2)	33
	Secondary+		222 (24.0)	18	191 (24.4)	30	182 (22.3)	46	118 (17)	39	61 (11)	49
Husband’s occupation	Farmer		634 (68.6)	15	538 (68.7)	47	577 (70.6)	35	528 (76)	35	449 (81.1)	36
	Employed		226 (24.4)	18	188 (24)	29	194 (23.8)	48.5	141 (20.3)	43	86 (15.5)	47
	Job seeker		64 (7.0)	16	57 (7.3)	38	46 (5.6)	37.5	26 (3.7)	34.5	19 (3.4)	43
Ideal number of children	< = 4	1056 (64.0)	478 (51.7)	15	415 (53)	40	397 (48.6)	38	302 (43.5)	37	195 (35.2)	38
	5+	593 (36.0)	446 (48.3)	15	368 (47)	41	420 (51.4)	35	393 (56.5)	35	359 (64.8)	36
Contraceptive ever use	Yes	623 (37.8)	573 (62)	15	499 (63.7)	39	509 (62.3)	37	430 (61.9)	36	328 (59.2)	37
	No	1026 (62.2)	351 (38)	15	284 (36.3)	51	308 (37.7)	36	265 (38.1)	36	226 (40.8)	36
Women empowerment	None	806 (48.9)	425 (46)	15	355 (45.3)	40	383 (48.9)	35	351 (50.5)	35	304 (54.9)	36
	Empowered	843 (51.1)	499 (54)	15	428 (54.7)	41	434 (53.1)	39	344 (49.5)	37	250 (45.1)	38
Wealth index	Lowest	351 (21.3)	180 (19.5)	15	136 (17.4)	40	169 (20.7)	35	158 (22.7)	34	139 (25.1)	36
	Lower	327 (19.8)	191 (20.7)	15	172 (22)	49.5	159 (19.5)	35	140 (20.1)	35	118 (21.3)	36.5
	Middle	325 (19.7)	216 (23.4)	14	189 (24.1)	43	200 (24.5)	35	182 (26.2)	37	154 (27.8)	36.5
	Higher	313 (19.0)	164 (17.7)	16	144 (18.4)	41.5	139 (17)	39	107 (15.5)	35	77 (13.9)	40
	Highest	333 (20.2)	173 (18.7)	18	142 (18.1)	28	150 (18.3)	53	108 (15.5)	46	66 (11.9)	46
Women marriage cohort	Before 1990		127 (13.74)	14	116 (14.8)	64	121 (14.8)	35	119 (17.1)	34	115 (20.7)	35
	1990–1999		280 (30.3)	14	241 (30.8)	54	270 (33)	36	266 (38.3)	36	247 (44.6)	39
	2000–2009		291 (31.5)	15	234 (29.9)	38	280 (34.3)	40	247 (35.5)	38	176 (31.8)	38
	2010–2019		226 (24.46)	18	192 (24.5)	28	146 (17.9)	32.5	63 (9.1)	24	16 (2.9)	23
**Total**			**924 (100)**	**15**	**783 (100)**	**35**	**817 (100)**	**36**	**695 (100)**	**36**	**554 (100)**	**36**

Median AFM* = Median Age at First Marriage

**Table 2:** Multivariate analysis of age at first marriage and birth intervals for ever-married women, in Dabat HDSSs, Ethiopia, 2020.

Characteristics	Categories	Models									
		Model 1		Model 2		Model 3		Model 4		Model 5	
		TR	95% CI	TR	95% CI	TR	95% CI	TR	95% CI	TR	95% CI
Current age	15–24	1.12***	1.06–1.18	1.02	0.76–1.37	1.01	0.80–1.26	1.26	0.87–1.84	1.41	0.65–3.10
	25–34	1.05**	1.02–1.10	1.17	0.97–1.41	1.02	0.89–1.15	1.04	0.89–1.22	1.17	0.97–1.41
	35+	1.00(ref)		1.00(ref)		1.00(ref)		1.00(ref)		1.00(ref)	
Age at first marriage	< 18			1,00(ref)		1.00(ref)		1.00(ref)		1.00(ref)	
	≥ 18			0.91	0.77–1.07	0.88*	0.78–0.99	0.95	0.81–1.13	1.05	0.85–1.28
Educational Level	None	1.00(ref)		1.00(ref)		1.00(ref)		1.00(ref)		1.00(ref)	
	Primary	1.05*	1.00–1.10	0.88	0.75–1.04	1.18**	1.05–1.32	1.02	0.88–1.18	0.97	0.81–1.16
	Secondary+	1.21***	1.14–1.28	1.04	0.83–1.31	1.57***	1.30–1.89	1.55***	1.23–1.97	1.18	0.83–1.68
Media exposure	Yes	0.99	0.93–1.05	1.09	0.88–1.34	1.02	0.85–1.21	0.91	0.71–1.19	1.51*	1.05–2.17
	No	1.00(ref)		1.00(ref)		1.00(ref)		1.00(ref)		1.00(ref)	
Occupation	Housewife	1.00(ref)		1.00(ref)		1.00(ref)		1.00(ref)		1.00(ref)	
	Student	1.53***	1.43–1.64	1.33	0.98–1.81	1.04	0.68–1.59	0.92	0.49–1.73	-	-
	Employed	1.23***	1.15–1.33	1.03	0.83–1.29	1.11	0.92–1.33	1.09	0.87–1.39	1.23	0.89–1.72
	Job seekers	1.32***	1.23–1.43	1.15	0.88–1.49	1.19	0.94–1.49	1.54	1.13–2.09	1.29	0.76–2.21
Divorce before 1^st^ birth	Yes			1.86***	1.62–2.15						
	No			1.00(ref)							
Divorce experience	Yes					1.24***	1.13–1.36	1.05	0.93–1.18	1.08	0.95–1.23
	No					1.00(ref)		1.00(ref)		1.00(ref)	
Child death experience	Yes			0.88	0.75–1.04	0.79***	0.71–0.89	0.81**	0.71–0.92	0.81**	0.69–0.94
	No			1.00(ref)		1.00(ref)		1.00(ref)		1.00(ref)	
Husband’s occupation	Farmer			1.00(ref)		1.00(ref)		1.00(ref)		1.00(ref)	
	Employed			0.85	0.66–1.10	1.07	0.87–1.33	1.13	0.88–1.47	1.17	0.83–1.67
	Job seeker			0.96	0.73–1.25	1.28*	1.01–1.62	0.95	0.70–1.29	1.16	0.80–1.70
Ideal children	< = 4	1.06**	1.03–1.10	1.00(ref)		1.00(ref)		1.00(ref)		1.14*	1,01–1.28
	5+	1.00(ref)		0.88*	0.79–0.99	0.91*	0.84–0.98	0.87**	0.78–0.96	1.00(ref)	
Women empowerment	None	1.03	0.99–1.07	0.86*	0.76–0.96	0.93	0.85–1.02	1.00(ref)		0.97	0.86–1.09
	Empowered	1.00(ref)		1.00(ref)		1.00(ref)		0.98	0.88–1.08	1.00(ref)	
Wealth index	Lowest	1.12***	1.07–1.18	1.06	0.89–1.25	0.95	0.73–1.24	0.87*	0.75–0.99	0.95	0.82–1.11
	Lower	1.04	0.99–1.09	1.23**	1.06–1.43	0.96	0.74–1.26	1.02	0.89–1.15	1.04	0.90–1.20
	Middle	1.00(ref)		1.10(ref)		0.91	0.70–1.18	1.00(ref)		1.00(ref)	
	Higher	0.95	0.87–1.03	1.09	0.83–1.42	0.93	0.77–1.12	0.90	0.71–1.13	1.05	0.81–1.36
	Highest	0.96	0.86–1.07	0.80	0.57–1.13	1.00(ref)		1.05	0.73–1.50	0.89	0.55–1.43
Women marriage cohort	Before 1990			1.33***	1.14–1.57	0.94	0.80–1.09	0.98	0.86–1.11	0.84*	0.74–0.97
	1990–1999			1.00(ref)		0.95	0.83–1.08	1.00(ref)		1.00(ref)	
	2000–2009			0.76**	0.63–0.92	1.00(ref)		1.03	0.88–1.21	0.89	0.74–1.09
	2010–2019			0.73*	0.54–0.98	1.00	0.85–1.17	1.08	0.79–1.45	0.83	0.49–1.38

Significance level

*** p<0.001

** p<0.01

* p<0.05; TR = Time Ratio

### Data processing and analysis

Data on women aged 15 to 49 years were entered into Open Data Kit (ODK) software suites and exported to STATA version 14 for cleaning and analysis. Descriptive statistics including frequencies and proportions were used to summarize the variables of interest. Median age at first marriage and duration of birth intervals were also carried out along with the respondents’ characteristics. The survival analysis methods were carried out because the study contained censored data. Log rank test was used to test the significance of different categorical factors under this study. The model selection in this study was done based on the distributional assumption of the baseline hazard. Based on this assumption, if the baseline hazard had a specific distributional pattern, comparison will be made among parametric survival models. In this study the baseline hazard had a distributional pattern. Akaike’s Information Criterion (AIC) was used for model comparison or to select the models, and the parametric survival models with low AIC value were considered as a best-fitted model. So, five models containing variables of interest were fitted for this study to determine the effects of various factors on age at first marriage and birth intervals. Model I examined the effects of predictor variables on age at first marriage. Models II, III, IV and V assessed the effects of predictors on first, second, third and fourth birth intervals, respectively. Based on AIC, the parametric survival models such as Loglogistic regression (AIC = 842.6) model for model I, Lognormal regression (AIC = 1676.72) for model II, and Loglogistic regression for Models III, IV and IV have the smallest AIC value (with AIC = 1260.43, 1243.01 and 1012.57 respectively) among the models considered. In parametric models, we measure the direct effect of the explanatory variables on the survival time instead of hazard. In this study, the effect size for parametric model is the time ratio. For each covariate, a time ratio value greater than one can be interpreted as individual experiences the event at a later timing. On the other hand, a time ratio value of less than one implies that individuals will experience the event of interest faster. Adjusted time ratio (ATR) with 95% Confidence interval in the multivariable model was used to select variables that have a statistically significant association with age at first marriage and birth intervals. Few cases pertaining to premarital births and shorter birth intervals (less than 7 months) were dropped. In this study, p-value less than 0.05 is considered to be statistically significant.

## Results

### Socio-economic and demographic characteristics

Out of 1683 women of reproductive age planned to be included in the study, 1649 have participated in the interview making respondent rate of 98%. The study revealed that 1062 (64.4%) respondents were from rural areas, and about 18% were born in urban areas. Women whose age less than 25, and greater than 34 years constituted 47% and 28.74% respectively. Among respondents, 35.4% had no formal education, and 47% of them were housewives in occupation. Among the participants, ever married, currently married and never married/single women accounted 924(56%), 812(49.2%) and 725(44%) respectively. Women who had media exposure and whose ideal number of children greater than four accounted 27.8% and 36% respectively. Among all women age 35–49, 94% have been married at least once which indicated that marriage is nearly universal in the study area (Table 1).

### Differentials in median length of age at first marriage

As mentioned elsewhere among participants (1649), 924 (56%) women have been married at least once in their lives. The results showed that there are significant variations in marriage by socioeconomic and demographic characteristics of women. Among ever married women, 303 (32.8%) were from urban areas, 22.9% had media exposure, and 15.7% & 48.3% women age less than 25 and greater than 34 years respectively. Among ever married women, 56.6% had no formal education, and 75.3% of them were housewives in occupation. Ever married women whose age at first marriage and birth less than 18 years accounted 71.6% and 41% respectively. Among husbands, 68.6% were a farmer in occupation and 58% had no formal education. The marriage cohort between 1990 and 1999, and 2000 and 2009 constituted 30.3% and 31.5% of ever married women respectively. The oldest marriage cohort (married before 1990) and the recent marriage cohort (married between 2010 and 2020) also accounted 13.74% and 24.46% respectively. Two hundred fifteen (23.3%) ever married women had divorce experience at least once in their lives, and of these, nearly two-third of them had divorced before they gave birth to their first child (Table 1).

Table 1 below also shows the median age at first marriage, MAFM, by socioeconomic and demographic characteristics of women. The overall MAFM in the study area was 15 years. It was highest for the employed (19) followed by job seekers (18), and lowest for housewives (15). Differentials by current age showed that the MAFM was higher (16) in the age less than 25, than women with 25 or more years (15). Regarding education, women with no formal education married 1 and 4 years earlier than those with primary, and secondary or more education respectively. The MAFM for those who had their first marriage and birth at 18 or more years were 5 and 3 years later than those who had their first marriage and birth less than 18 years respectively. It was higher in urban women (18) than in rural women (15), and higher in women who had media exposure (18) than those who didn’t have (15). The MAFM was higher among women whose husbands were employed and secondary or above educational status. Women with better wealth status and recent marriage cohort had higher MAFM (Table 1).

### Differentials in median length of birth intervals

Among ever married women (924), 836 (90.5%), 717 (77.6%) and 572 (61.9%) had at least first, second and third birth respectively. Among those, 783, 817, 695 and 554 women were included in the interval analyses by dropping 141, 19, 22 and 18 women who had shorter birth intervals (less than 7 months) respectively. Never married women who gave birth to a child accounted 5.6% (41) and majority of them (34) lived in urban areas. Those women who have a premarital birth were removed from the analysis because the duration between first marriage and first birth is considered first birth interval. Other intervals are calculated by the difference between two successive live births after marriage.

Table 1 below shows that the median first, second, third and fourth birth intervals do not differ much. Married women seem to wait a median duration of almost three years for their first, second, third and fourth child. However, the results displayed that there are substantial differences in median birth intervals by selected socioeconomic and demographic characteristics of women.

#### First birth interval

The median interval between first marriage and first birth (first birth interval) was longer in rural areas (43 months) compared to those in urban areas (36 months). The median duration of first birth interval of women who married before 18 years (47 months) was greater than those who married at age 18 or more years (28 months). It was longer for women with no formal education (51.5 months) but shorter for those whose educational status was secondary or above (30 months). There was a consistent increase in first birth interval from younger (28 months) to older (51 months) age groups. First birth interval for women who had media exposure was shorter (32 months) than those who didn’t have (43 months). Housewives had longer (43 months) first birth interval than employed women (30 months). Women who had divorce experience before first birth had longer first birth interval (67 months) than those who didn’t have (37 months). Women who had child death experience, whose husbands were farmers and with no formal education had longer first birth intervals, and women with highest wealth status had shorter birth intervals. There was a steady decline in first birth interval from older (64 months) to younger (28 months) marriage cohorts (Table 1).

#### Second, third and fourth birth intervals

Contrary to first birth interval; women in urban areas, employed in occupation, had media exposure, with no child death experience and with secondary and above educational status had longer second, third and fourth birth intervals. In addition, women whose husbands were employed in occupation and with secondary or more education, and those women with highest wealth status had longer second, third and fourth birth intervals.

### Average duration of birth interval, exclusive breastfeeding and postpartum amenorrhea in three years preceding the survey, 2020

**Fig 1:**
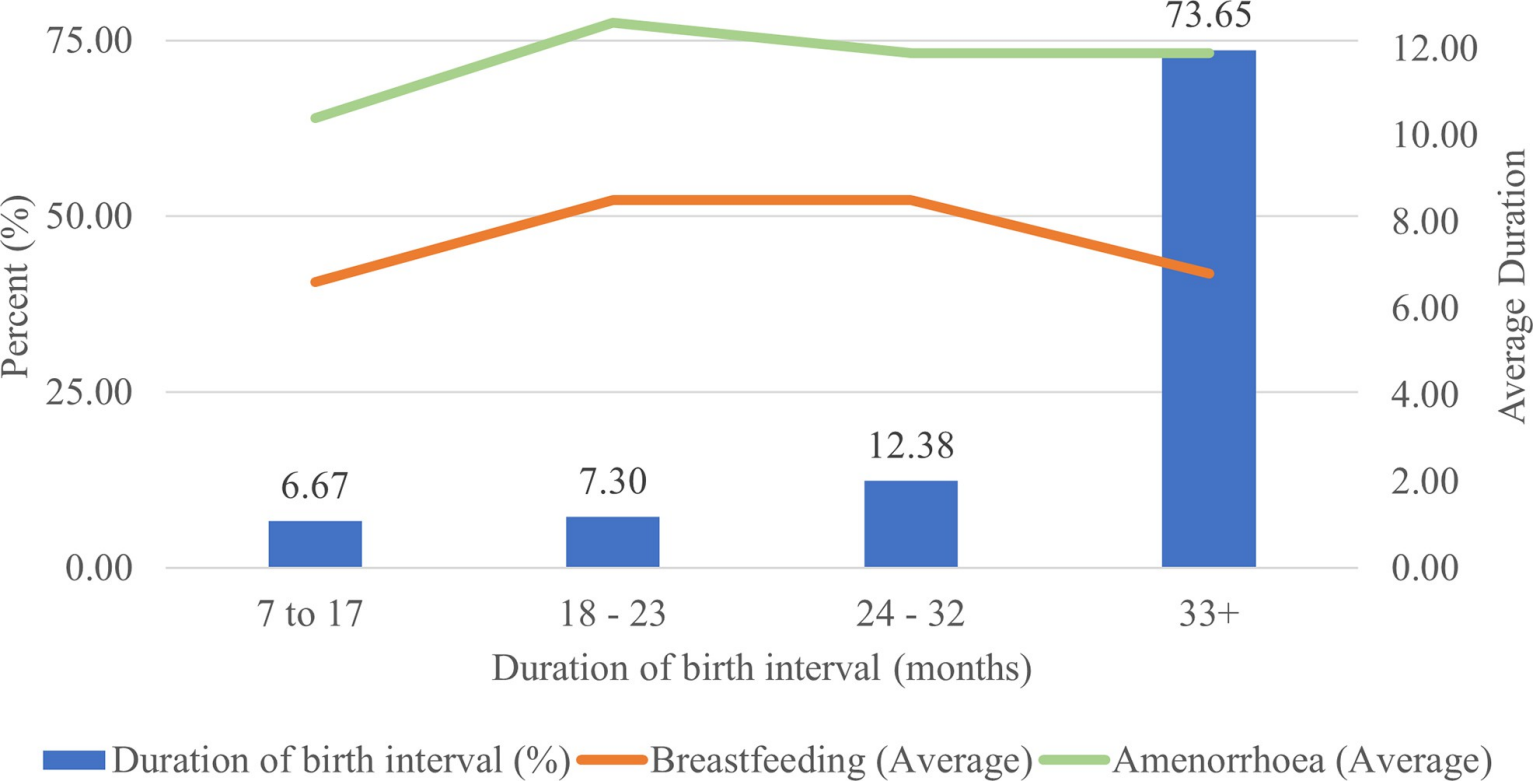
Percentage of women by duration of birth intervals, average exclusive breastfeeding and average postpartum amenorrhea during three years preceding the survey.

In three years preceding the 2020 survey, 443 children were born to 394 mothers. Among 394 women, 88.6%, 10.4% and 1% gave one birth, two births and three births, respectively. On the other hand, from 443 births, 79 births didn’t have a birth interval for 78 children were first birth order and one twin second birth order. Thus, the birth interval analysis in three years preceding the survey was computed for 315 mothers. Duration of birth interval considered was the time between their most recent birth and the one preceding the last birth. Hence, among these women, 6.67%, 7.3%, 12.38% and 73.65% gave birth within an interval duration of 7–17 months, 18–23 months, 24–32 months, and 33 & above months, respectively. The mean and median birth intervals of women who gave birth in three years preceding the survey were 53.88 and 46 months, respectively (Fig 1).

The average duration of exclusive breastfeeding and postpartum amenorrhea had shown a slight increase as the birth interval elongates beyond 17 months. While the average duration of amenorrhea remained stable for a birth interval of more than 18 months duration, the average duration of breastfeeding was relatively shorter for the longest birth interval category as compared to births of 18–32 months of birth interval. The mean and median duration of exclusive breastfeeding and postpartum amenorrhea of women who gave birth in three years preceding the survey were 7 and 6 months, and 11.6 and 12 months respectively (Fig 1).

### Determinants of the timing of first marriage and birth intervals: Multivariate analysis

For time-to-marriage and birth interval data, multivariable parametric models of log-logistic and log-normal distribution were fitted. Table 2 shows the summarized results of parametric models. A total of 1649 women with reproductive age were included in the survival regression analyses of the timing of age at first marriage. In addition, 783, 817, 695 and 554 ever married women were included for analysis of first, second, third and fourth duration of birth intervals and their predictors. Variables such as residence, age at first birth, husband’s education and contraceptive ever use that didn’t show any association in either of the outcome variables in the models are excluded from Table 2.

### Timing of age at first marriage

The Log-logistic regression analysis (model 1) shows that current age of women, educational level, occupation, ideal number of children, and wealth status of women have a statistically significant relationship with timing of first marriage. According to the result, younger women have slower transition to first union compared to older women. Woman’s educational attainment significantly delayed the timing of marriage. Women with primary (TR = 1.05, CI = 1.00–1.10) and secondary (TR = 1.21, CI = 1.14–1.28) education had slower transition to first marriage compared to those without any formal education. Compared to housewives in occupation, students (TR = 1.53, CI = 1.43–1.64), employed women (TR = 1.23, CI = 1.15–1.33) and job seekers (TR = 1.32, CI = 1.23–1.43) wait longer time before first marriage. Women who had less than or equal to four ideal number of children wait longer time before first marriage compared to those with five or more ideal number of children (TR = 1.06, CI = 1.03–1.10). Women with the lowest wealth status postponed marriage timing compared to those with middle status (TR = 1.12, CI = 1.07–1.18).

### Duration of birth intervals

Based on the Log-normal model (model 2), divorce experience before first birth, ideal number of children, women empowerment, and marriage cohort of women were significant predictors of the duration of first birth interval. Women who had divorce experience before first birth have longer first birth interval than those who didn’t have divorce experience (TR = 1.86, CI = 1.62–2.15). Women with five or more ideal number of children had faster transition to first birth after marriage compared to those with less than or equal to four ideal number of children (TR = 0.88, CI = 0.79-.99). Women who hadn’t empowered to decide the households’ monthly expense have shorter first birth interval than those who had empowered (TR = 0.86, CI = 0.76–0.96). Compared to the middle wealth status, women with lower status have longer first birth intervals (TR = 1.23, CI = 1.06–1.43). Women with the oldest marriage cohort, married before 1990, have longer first birth interval than those women who got married between 1990 and 1999 (TR = 1.33, CI = 1.14–1.57). First birth interval was also shorter among women who got married between 2000 and 2009 (TR = 0.76, CI = 0.63–0.92) and after 2009 (TR = 0.73, CI = 0.54–0.98) compared to those who got married between 1990 and 1999. Women who are in the older marriage cohort waited longer to have their first child after first marriage.

The Log-logistic model (model 3) shows that age at first marriage, women education, divorce experience, child death experience, and ideal number of children have significant effects in the second birth interval, duration between first and second births. Women whose age at first marriage was greater than 18, legal age, have faster transition to second birth (TR = 0.88, CI = 0.78–0.99). The interval between first birth and second birth was longer among women who spent some time in schooling (primary, TR = 1.18: CI = 1.05–1.32, & secondary and above, TR = 1.57; CI = 1.30–1.89) than those who didn’t have formal education. Women who had divorce experience in their lives have longer second birth interval (TR = 1.24: CI = 1.13–1.36) than who didn’t have, and it is shorter among women who had child death experience (TR = 0.79: CI = 0.71–0.89) compared to those who didn’t have the experience. Women who had five or more ideal number of children had shorter second birth interval compared to those with less than or equal to four ideal number of children (TR = 0.91, CI = 0.84-.98).

Model 4 in Table 2 (Log-logistic regression) also shows that women education, child death experience, ideal number of children and wealth status of women have significant effect in the third birth interval. Compared to women with no formal education, those with secondary or more educational status have longer third birth interval (TR = 1.55, CI = 1.23–1.97). The fourth birth interval is shorter among women who had child death experience (TR = 0.81: CI = 0.71–0.92) compared to those who didn’t have the experience. Women who had five or more ideal number of children had shorter second birth interval compared to those with less than or equal to four ideal number of children (TR = 0.87, CI = 0.78–0.96).

In the case of the fourth birth interval (model 5), Log-logistic regression model illustrates that media exposure, child death experience, ideal number of children and marriage cohort of women are correlated with the fourth birth interval. Compared to women who didn’t have media exposure, those who had media exposure have longer fourth birth interval (TR = 1.51, CI = 1.05–2.17). The fourth birth interval is shorter among women who had child death experience (TR = 0.81: CI = 0.69–0.94) compared to those who didn’t have the experience. Women who had less than or equal to four ideal number of children had longer fourth birth interval compared to those with five or more ideal number of children (TR = 1.14, CI = 1.01–1.28), and it is shorter among women who got married before 1990 compared to those who got married between 1990 and 1999 (TR = 0.84, CI = 0.74–0.97).

## Discussion

This study is conducted to analyse the covariates of age at first marriage and birth spacing and to examine their effects on the current fertility. The result showed that the population in the study area was characterized by low median age at first marriage and longer successive birth intervals. The age at first marriage was estimated at 15 years which was below the national and regional average [8]. The existence of variations in socio-cultural norms, values, and traditions encourages early marriage in Amhara region compared to other parts of the country [25]. This could be due to the desire of the family to ensuring virginity of girls upon marriage to keep parents’ social esteem and to serve family kinship [9]. However, the median age at first marriage was found to be higher for women in urban areas, younger age group, recent marriage cohort, better educated, employed and highest wealth status which is consistent with those of earlier studies conducted at regional and national levels in the country [8, 26–28].

The result of the study also revealed that married women waited almost a median duration of three years for their first child with median age at first birth of 18 years. The median interval between first marriage and first birth, first birth interval, is nearly equal to that of in Amhara region which was the highest among other regions in the country [8]. This may be the fact that as marriage was very early, young married women neither biologically ready to become pregnant nor psychologically prepared to be a mother [28]. Besides, young married women may spend more time in their parents’ and/or fathers-in-law’s home before they have their first child. The other reason maybe they may not know one another leading to divorce that takes longer time to give birth to their first child after remarriage, in this study nearly two third of the divorces taken place before first birth. For all these reasons, first birth interval in the study area is longer. This result may be partly explaining the question of why fertility level in Amhara region is low compared to other regions while age at first marriage is still low. However, the result indicated that there are substantial differences in median first birth interval by selected socioeconomic and demographic characteristics of women. The median length of first birth interval after marriage decreases with urban residents, higher age at marriage, better educational attainment, wealth status, employment status and media exposure. Women who married at a relatively later time had a significantly shorter timing to first birth.

On the other hand, the result revealed that married women waited a median duration of three years for their second, third and fourth child. However, contrary to first birth interval, the median length of second, third and fourth birth intervals were longer among women of urban residents, high age at marriage, better educational attainment, wealth status, employment status and media exposure. Women with these background characteristics got married at later age but not waiting longer for the first baby after marriage but delaying the second, third and fourth births by spacing the consecutive birth intervals.

The median birth interval of married women for three years preceding the survey was also estimated at 46 months (3.8 years) which was higher than the previous studies [8, 15, 29, 30] and higher than the recommendation for birth interval by WHO [20].

Results of multivariate analyses indicate that current age of women, educational level, occupation, ideal number of children, and wealth status of women have a statistically significant relationship with timing of first marriage. Younger women took longer time to get married than those who are higher ages. It may be partly attributed to younger women may have the chance to have higher educational attainment compared with their older counterparts. In addition, since recent times, marriage among younger women is based on self-selection of a partner that may contribute for late marriage. This finding is consistent with those of earlier studies conducted on Ethiopian women [4, 5]. Education level was a significant factor in age at first marriage. Females with primary and above education waited longer time to marry than those with no education which is consistent with previous studies [25, 31] This may be the fact that educated women spend a longer time at school and they may have the chance for labore force participation outside home. Even educated women may know well the risk of getting marriage early.

The types of occupations in which women were engaged also affected the age at first marriage. Employed women waited longer time before first marriage compared to housewives. This may be the fact that employed women were possibly educated and were likely to marry at later ages. Employment influences on women’s marital decisions as it tends to lead women to appreciating singlehood and cohabitation over marriage [32]. On the other hand, job seekers usually postpone their marriage until they get their own job. The result also revealed that students stayed longer before first marriage. This may be the fact that girls who stay in school longer have a direct influence to postpone their probability of having married [33].

Women with the lowest wealth status postponed marriage timing compared to those with middle status which is consistent with studies in Ethiopia [25, 34] and in Tanzania [35]. Since recent time, in developing countries, poor people have tended to have fewer number of births as compared to their wealthy compatriots. This might be attributed to the increasing severe economic difficulties that may initiate women not to give birth. The result also revealed that women who had lower number of ideal number of children wait longer time to get marriage. This may be the fact that women who need to have large number of children in the future need to marry very early to achieve the desired number of children.

The parametric survival analysis also identified the variables that were statistically associated with the duration of first, second, third and fourth birth intervals. Women who had divorce experience before first birth have longer first birth interval. This could be the fact that a woman who already divorced before her first birth may take longer time to give birth to her first child after remarriage. The result also showed that women with five or more ideal number of children had shorter first, second and third birth intervals. This may be women who desire to have large number of children in the future need to give birth to a child immediately after marriage or a birth to achieve the desired number of children which is in line with the previous studies [18]. Women who didn’t have power to decide the households’ monthly expense have shorter first birth interval than those who had empowered. This might be attribute to the fact that women who had this decision-making power may decide on households’ economic resources that enable them to use contraceptive methods which is consistent with previous studies [36, 37].

The study also revealed that women with the oldest marriage cohort waited longer time to give birth to first child after marriage compared to those with recent marriage cohort, consistent with a study conducted in Amhara region [27] and China [4]. This may be attributed to the low age at first marriage, those women with the oldest marriage cohort got married early. Women who got married early are neither biologically ready to become pregnant nor psychologically prepared to be a mother. Besides, Women may spend more time in their parents’ and/or fathers’-in-law home before they have their first child, and the young couples may not know one another leading to divorce that takes longer time to give birth to their first child after remarriage. Even previous studies confirmed that the relatively low health conditions and nutritional levels of women in the past may be the reason for the lesser fecundity of women which in turn delay the first birth after marriage [1].

Women’s higher educational attainment is associated with longer second and third birth intervals compared to women without formal education. This might be attribute to the fact that when the education status of women increased, knowledge and awareness of the women upon the consequences of short birth interval on maternal and child health and the benefit of small family size will also be enhanced. This is similar to other studies [18, 38]. Women who had exposure to any media have longer fourth birth interval compared to those who didn’t have media exposure. Women who have exposure to any media are expected to have a better understanding of the negative impact of short birth interval on family size and even on maternal and children’s health which is consistent with other studies [18, 25]. Women who had child death experience had shorter second, third and fourth birth intervals compared to those who didn’t have the experience. This may be the fact that women who had a child-death experience were likely to have a higher number of children for replacement which is consistent with other studies [39, 40].

The effect of contraceptive use was not controlled for, as the interval was computed for some times in the past whereas information on use of contraceptive referred to the survey period. On the other hand, some of the findings contradict the results of previous studies in developing countries. Women’s residence was not statistically significant with either of the dependent variables, timing of age at first marriage and duration of birth intervals. The role of health extension workers in rural areas may help to have similar information with urban residents about the benefit of having desired number of children in a household. Age at first birth was the other variable that didn’t show any effect on the duration of birth intervals. This may be the fact that women who gave first birth at later age may have successive births until they achieve the desired number of children.

## Conclusion

The current research result showed that the median age at first marriage was the lowest which was below the national and regional average. However, the population in the study area was characterized by longer successive birth intervals with almost a median duration of three years for the first, second, third and fourth child, and even the longest median birth interval for three years preceding the survey (the recent birth interval) that harmonizes the effect of low age at first marriage for fertility rate. Longer successive birth interval is likely to reduce the total fertility rate of a woman. Increasing duration of successive birth intervals directly affect completed fertility by reducing the number of years available for childbearing of a woman. Women who delay child bearing may consequently have low fertility.

The parametric survival analysis also identified that women’s educational attainment and occupation contributed most for the timing of age at first marriage. In addition, it is also found that the decision to give birth to a child at a given interval depends on women’s educational attainment, child death experience and ideal number of children a woman would have. Hence, encouraging women for higher education and giving opportunity to women in employments rather than housewives, working at home, which may decrease the child mortality and shape the desired number of children, may be the most influential way of delaying age at first marriage and increasing the duration of successive birth intervals which in turn slowing down the fertility of women in the study area.

## Supporting information

S1 Data (DTA)

## Abbreviations

HDSSs: Health and Demographic Surveillance Site
TFR: Total Fertility Rate
ANRS: Amhara National Regional State
MAFM: Median Age at First Marriage
